# Establishing SARS-CoV-2 membrane protein-specific antibodies as a valuable serological target via high-content microscopy

**DOI:** 10.1016/j.isci.2023.107056

**Published:** 2023-06-07

**Authors:** Daniel M. Williams, Hailey R. Hornsby, Ola M. Shehata, Rebecca Brown, Marta Gallis, Naomi Meardon, Thomas A.H. Newman, Megan Plowright, Domen Zafred, Amber S.M. Shun-Shion, Anthony J. Hodder, Deepa Bliss, Andrew Metcalfe, James R. Edgar, David E. Gordon, Jon R. Sayers, Martin J. Nicklin, Miles Carroll, PITCH Consortium, Paul J. Collini, Stephen Brown, Thushan I. de Silva, Andrew A. Peden

**Affiliations:** 1 School of Bioscience, University of Sheffield, Western Bank, Sheffield S10 2TN, UK; 2 Department of Infection, Immunity and Cardiovascular Diseases, University of Sheffield Medical School, Beech Hill Road, Sheffield S10 2RX, UK; 3 Department of Pathology, University of Cambridge, Cambridge CB2 1QP, UK; 4 Department of Pathology, Emory University, Whitehead Building, Atlanta, GA, USA; 5 South Yorkshire Regional Department of Infection and Tropical Medicine, Sheffield Teaching Hospitals NHS Foundation Trust, Glossop Road, Sheffield S10 2JF, UK; 6 Wellcome Centre for Human Genetics, Nuffield Department of Medicine, University of Oxford, Oxford OX3 7BN, UK

**Keywords:** Biological sciences, Immunology, Microbiology, Cell biology

## Abstract

The prevalence and strength of serological responses mounted toward SARS-CoV-2 proteins other than nucleocapsid (N) and spike (S), which may be of use as additional serological markers, remains underexplored. Using high-content microscopy to assess antibody responses against full-length StrepTagged SARS-CoV-2 proteins, we found that 85% (166/196) of unvaccinated individuals with RT-PCR confirmed SARS-CoV-2 infections and 74% (31/42) of individuals infected after being vaccinated developed detectable IgG against the structural protein M, which is higher than previous estimates. Compared with N antibodies, M IgG displayed a shallower time-dependent decay and greater specificity. Sensitivity for SARS-CoV-2 seroprevalence was enhanced when N and M IgG detection was combined. These findings indicate that screening for M seroconversion may be a good approach for detecting additional vaccine breakthrough infections and highlight the potential to use HCM as a rapidly deployable method to identify the most immunogenic targets of newly emergent pathogens.

## Highlights

• HCM can detect SARS-CoV-2 N and S IgG responses with high specificity and sensitivity
• SARS-CoV-2 M antibodies are a third high seroprevalence marker (85% of patients with COVID-19)
• Anti-M IgG often displays a shallower time-dependent decline than N IgG post infection
• Screening for SARS-CoV-2 M antibodies alongside N can boost serological sensitivity

## Graphical abstract

**Figure:**
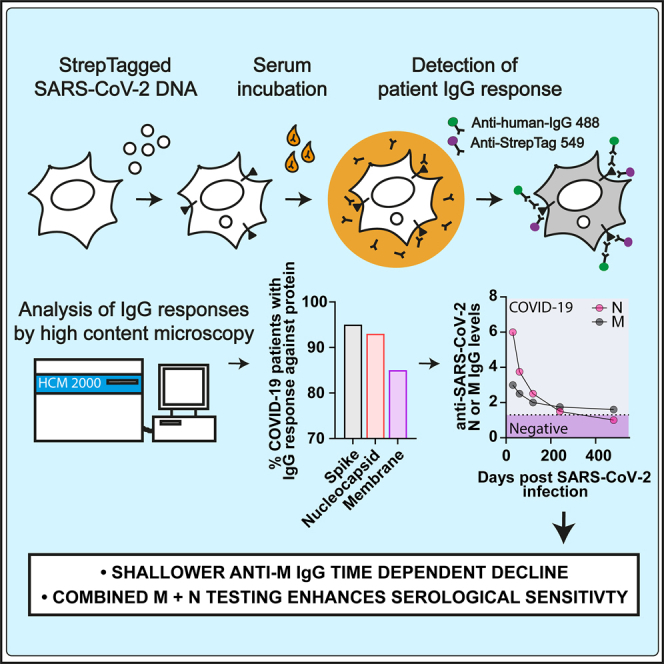

## Introduction

Controlling severe acute respiratory syndrome coronavirus 2 (SARS-CoV-2) infections within communities and across populations requires accurate knowledge of both current and previous SARS-CoV-2 infections. Serological assays fulfill critical roles toward the latter, identifying individuals previously exposed to the virus who may potentially be immune, while also contributing toward the construction of accurate epidemiological estimates of infection rates using serosurveys. Analysis of COVID-19 patient serum obtained through serological sampling has also provided invaluable information on the kinetics and profile of antibody responses produced against SARS-CoV-2 in relation to disease outcomes.^1^^,^^2^^,^^3^

To date, serological testing for SARS-CoV-2 relies upon detection of antibodies against either the nucleocapsid (N) or spike (S) proteins as both induce detectable humoral immune responses in the vast majority of those infected.^4^ Given its relative simplicity and high sensitivity, ELISA-based screening for reactivity of patient sera against purified versions of the N and S proteins is widely employed and is considered the gold standard for identification of patients who have previously been infected with SARS-CoV-2.^5^^,^^6^ However, the production of purified proteins for use in an ELISA can be costly and time consuming, with this same requirement also limiting the ability to assess antibody responses against other SARS-CoV-2 proteins whose physiochemical properties may be incompatible with production in a purified form.

In combination with immunofluorescence microscopy or flow cytometry, the use of mammalian cells as vehicles to express and present viral antigens to screen patient sera for antibodies can bypass the need for purified proteins and provide additional advantages over ELISA-based serological testing. Viral proteins can be easily transfected into mammalian cells^7^^,^^8^ and once expressed are then produced in a state which incorporates many features that form important parts of epitopes recognized by circulating antibodies, such as posttranslational modifications.^9^^,^^10^ Screening of serum samples by immunofluorescence microscopy demonstrated high sensitivity and specificity when applied during the SARS-CoV and MERS outbreaks^11^^,^^12^^,^^13^ but this methodology has largely been overlooked for use in SARS-CoV-2 serological studies.^14^^,^^15^^,^^16^^,^^17^^,^^18^

In this paper, we describe the development of a cell-based expression platform combined with high-content immunofluorescence microscopy (HCM) to identify the presence of antibodies to specific SARS-CoV-2 proteins and test its performance in comparison to ELISA-based serological testing. Calibration of our automated system using StrepTagged SARS-CoV-2 N and S proteins demonstrated that automated immunofluorescence-based antibody screening could detect N and S antibodies in sera collected from patients with COVID-19 with high sensitivity and specificity. Most interestingly, further application of this system identified antibodies against the SARS-CoV-2 membrane (M) protein in a significant number of RT-PCR-confirmed SARS-CoV-2-positive cases, including in individuals infected following vaccination. By tracking N, S, and M IgG levels measured at multiple time points post infection in the same individuals, we go on to characterize the kinetics of the anti-M antibody response relative to N and find that M IgG can in some cases be more durable than N IgG which wanes rapidly following COVID-19.

## Results

### Detection of SARS-CoV-2 antibodies using immunofluorescence microscopy

**Figure 1:**
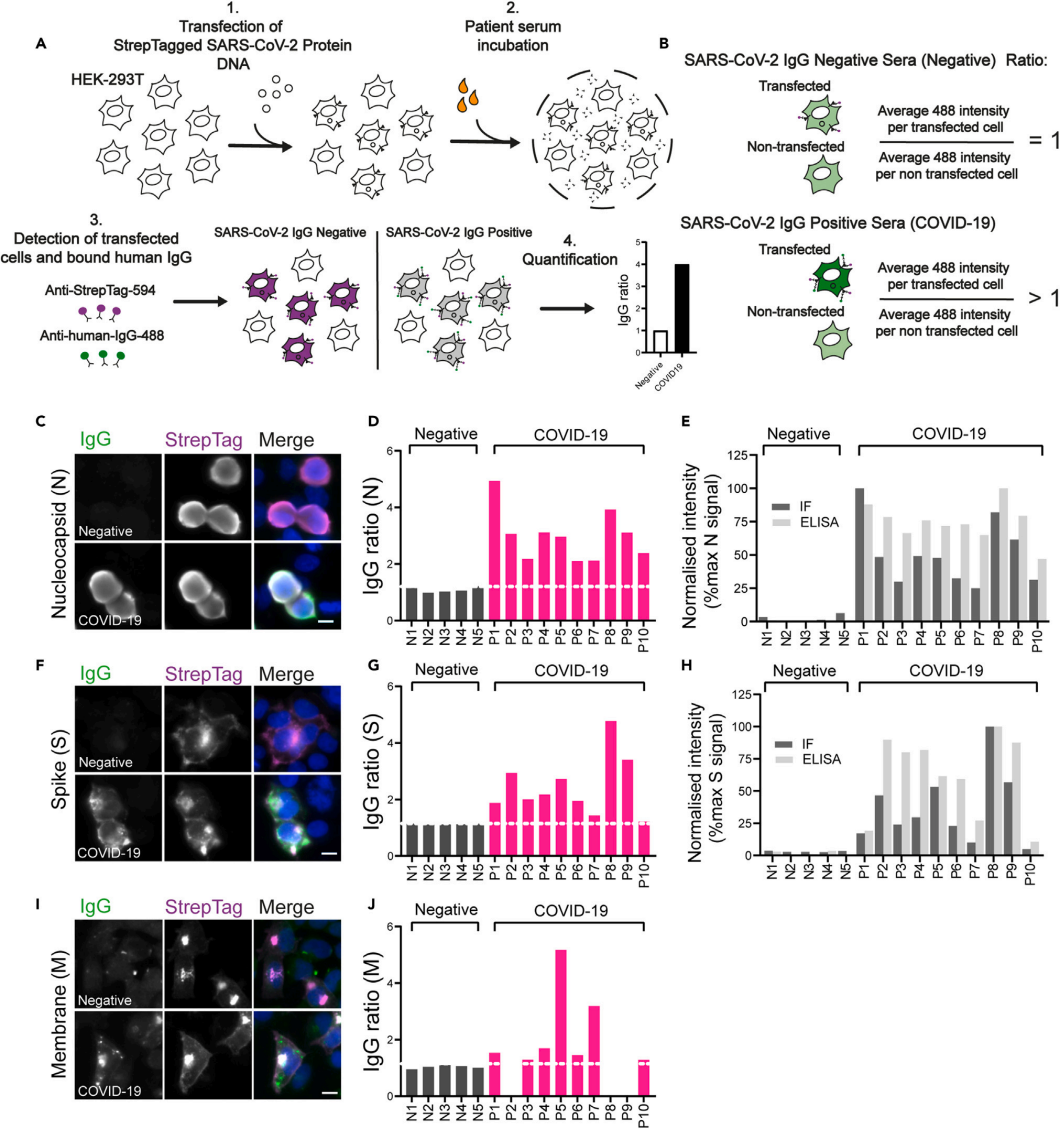
Detection of antibodies against SARS-CoV-2 proteins using immunofluorescence microscopy (A) Schematic of the workflow used to screen patient sera for SARS-CoV-2 antibodies by immunofluorescence microscopy. (B) Overview of how fluorescence intensity ratios used to estimate the relative strength of IgG responses against the indicated SARS-CoV-2 protein were calculated. Representative immunofluorescence microscopy images of HEK-293 cells transiently expressing StrepTagged SARS-CoV-2 (C) N, (F) S, or (I) M incubated with either pre-pandemic (Negative) or SARS-CoV-2-positive (COVID-19) patient sera. Bound human IgG and cells expressing StrepTag-Spike were detected with an Alexa 488 labeled anti-human IgG secondary antibody and StrepTactin-549, respectively. Quantification of Alexa 488 labeled anti-human IgG signal intensity associated with cells transfected with either (D) StrepTagged SARS-CoV-2 N, (G) StrepTagged SARS-CoV-2 S, or (J) SARS-CoV-2 M for pre-pandemic negative sera (N1-N5) and sera collected from RT-PCR confirmed SARS-CoV-2 cases (P1-P10). Comparison of (E) N and (H) S IgG responses measured by immunofluorescence with values generated by ELISA for the same samples. Due to limited amounts of sera, it was not possible to analyze all samples for M. Thus, some samples have been left blank in panel (J). Scale bars = 10 μm.

To test the effectiveness of immunofluorescence microscopy to detect antibodies against specific SARS-CoV-2 proteins in human plasma samples (Figures 1A and 1B), we transfected HEK-293T cells with codon-optimized plasmids encoding StrepTagged SARS-CoV-2 N and S (Figures 1C and 1F). Based on reports of antibody responses directed toward other coronavirus M proteins,^19^^,^^20^^,^^21^^,^^22^^,^^23^^,^^24^^,^^25^^,^^26^ we transfected cells with a StrepTagged SARS-CoV-2 M construct to determine if SARS-CoV-2-infected individuals also develop antibodies to M (Figure 1I). Following transfection, we performed a standard immunofluorescence labeling protocol with plasma obtained prior to the SARS-CoV-2 pandemic (pre-pandemic negative control samples) or from individuals confirmed to have been infected with SARS-CoV-2 by RT-PCR. The amount of fluorescent signal was quantified using ImageJ and a ratio between the non-transfected and transfected cells was calculated. As expected, the pre-pandemic samples produced IgG ratios close to 1.0 indicating low levels of cross-reactivity with the SARS-CoV-2 proteins. However, the samples collected from PCR-positive individuals showed IgG ratios greater than 1.0 indicating the presence of antibodies to the N and S proteins (Figures 1D and 1G). In addition, many samples also had detectable levels of IgG against the SARS-CoV-2 M protein (Figure 1J). In support of these observations, similar trends could be seen among ELISA readings, immunoblotting signals of N, and immunofluorescence IgG ratios (Figures 1E, 1H, and S2A–S2C). These results confirm that the combined use of transiently expressed tagged SARS-CoV-2 proteins with immunofluorescence microscopy is a viable approach to detect antibodies against SARS-CoV-2 antigens without the need for purified viral proteins.

### An automated microscopy pipeline can be used to quantify the levels of antibodies to SARS-CoV-2 proteins

**Figure 2:**
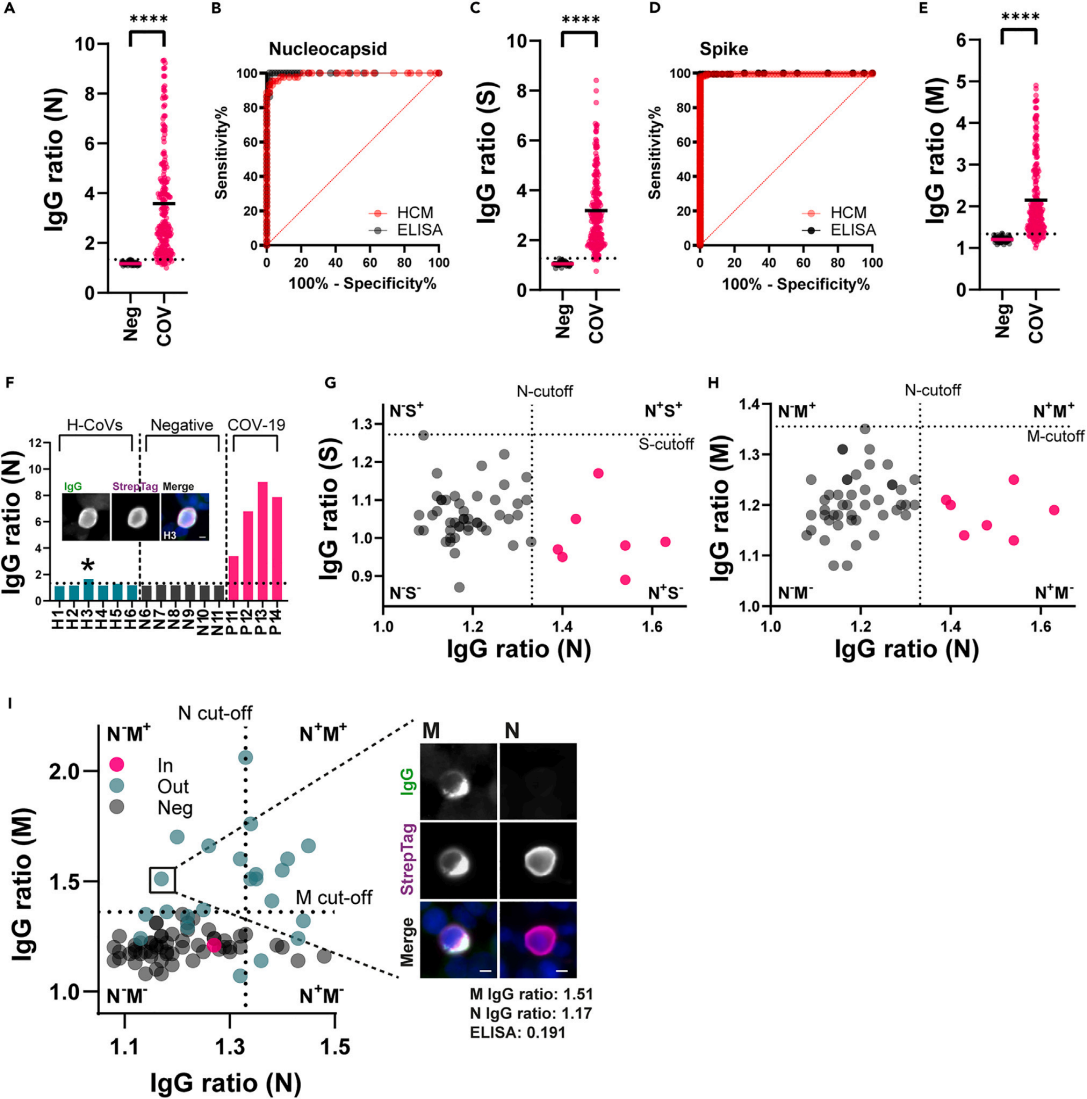
High-content microscopy and automated image analysis demonstrates high sensitivity and specificity for detection of SARS-CoV-2 N, S, and M IgG To assess the performance of HCM to identify SARS-CoV-2 antibodies against N, S, and M across a larger set of serum samples, HEK293-T cells were transfected with the indicated StrepTagged SARS-CoV-2 constructs and incubated with patient sera from a sample set consisting of 62 COVID-19-negative pre-pandemic and 196 COVID-19-positive samples. Bound IgG was detected with an anti-human Alexa-Flour488 conjugated secondary antibody and imaged by automated high-content immunofluorescence microscopy. Comparison of IgG ratios for pre-pandemic negative control samples and COVID-19-positive serum samples for (A) N (C) S and (E) M. ROC curves comparing the sensitivity and specificity of IF and ELISA-based detection of (B) N and (D) S IgG in SARS-CoV-2-positive and pre-pandemic sera. To investigate cross-reactivity of SARS-CoV-2 N with antibodies induced by exposure to seasonal coronaviruses (H-CoVs), a small number of samples taken from patients infected with seasonal H-CoVs (H1-H6) were incubated with cells expressing StrepTagged SARS-CoV-2 N (F). One sample (H3, highlighted with an asterix) had IgG signal to N which was above the threshold. Inset in (F) shows representative images for this sample. Scale bar = 5 μm. To explore whether sera from our pre-pandemic sample set showed any similar cross-reactivity with SARS-CoV-2 N, we plotted the IgG ratios for N from these samples against the IgG ratios measured for (G) S and (H) M in the same samples. Samples highlighted in pink represent sera that is positive for N IgG but negative for either SARS-CoV-2 S or M IgG suggesting cross-reactivity of antibodies induced against a seasonal coronavirus with SARS-CoV-2 N. (I) To determine if there were samples collected from patients with COVID-19 which were positive for M but lacked significant N reactivity, we plotted N IgG ratios from samples with low N antibody levels against M IgG ratios from the same samples. Region highlighted from the boxed data point shows representative images of IgG reactivity from this sample with M-StrepTag and N-StrepTag. Scale bar = 5 μm. ∗∗∗∗p < 0.0001 (Unpaired t-test).

To enhance the throughput of serological testing by immunofluorescence microscopy, we used a high-content microscope in conjunction with automated image analysis software (HCM) (Figures S3A–S3D) and repeated the experiments shown in Figure 1 using HCM. IgG ratios calculated automatically from images captured by HCM were comparable to those generated through manual quantification of the same samples (Figures S3E–S3G), demonstrating that the automated image capture and analysis workflow can accurately identify and quantify bound human IgG in plasma from SARS-CoV-2-infected individuals. To further test the platform and compare its performance to ELISA, we analyzed N and S IgG levels in 258 samples containing plasma from 62 individuals acquired prior to the SARS-CoV-2 pandemic (pre-pandemic negative controls) and 196 patients confirmed to have been infected with SARS-CoV-2 by RT-PCR. Using HCM, we observed a statistically significant difference in signal intensity for N and S antibodies between pre-pandemic and SARS-CoV-2-positive samples (Figures 2A and 2C). As previously reported,^27^ we found that IgG levels were significantly elevated for N and S in patients hospitalized after SARS-CoV-2 infection (inpatient) compared to non-hospitalized individuals (outpatient), with these trends also seen in ELISA absorbances for the sample sets (Figures S4A, S4B, S4E, and S4F). ELISA-based detection of N or S IgG in SARS-CoV-2-positive samples had marginally higher sensitivity than HCM when specificity was fixed at 100% (Figure 2B, N ELISA (185/196, 94.4%) vs. N HCM (182/196, 92.9%), Figure 2D, S ELISA (195/196, 99.5%) vs. S HCM (192/196, 98.0%)), Tables S1 and S2) with a greater number of samples from COVID-19 inpatients containing detectable N or S IgG (Figures S4C, S4D, S4G, and S4H, Tables S1 and S2). HCM-based serological testing can therefore provide a high level of specificity and sensitivity in detection of both SARS-CoV-2 N and S IgG, albeit slightly below the sensitivity provided by ELISA-based N and S testing.

### HCM reveals a significant proportion of patients with COVID-19 have IgG against the SARS-CoV-2 M protein

As a high percentage of SARS-CoV-2-infected samples in our training set had antibodies against the SARS-CoV-2 M protein, we also tested our larger sample set to further characterize the prevalence of anti-M antibody responses. As suggested from our pilot experiments, we observed a significant difference in anti-M signal intensity between the pre-pandemic SARS-CoV-2 negative samples and samples from RT-PCR-confirmed SARS-CoV-2-positive individuals (Figure 2E). Similar to N and S, our automated platform detected higher M antibody levels in COVID-19 inpatients (Figure S4I, Tables S1 and S2). ROC curve analysis of the above results (Figure S4J) established an optimal IgG ratio threshold of 1.36 for classification of M IgG ratios as positive or negative, providing 100% specificity and 84.7% (166/196) sensitivity. The percentage of samples with M IgG responses detected by HCM is higher than could be inferred from other recent studies^21^^,^^22^^,^^26^ and suggests M may be of use as a high-prevalence serological marker of SARS-CoV-2 infection.

### Detecting M can help reduce the number of false positives detected by N-based serological assays

While establishing our serological platform, we found multiple instances of above threshold N IgG signal in pre-pandemic plasma samples, including some that were collected from individuals with RT-PCR-confirmed seasonal human coronavirus infections (Figures 2F–2H). Each of these samples contained no detectable antibodies against either SARS-CoV-2 S or M (Figures 2G, 2H, S5A, and S5B). Consistent with the results seen by HCM, several of the pre-pandemic samples positive for N IgG by HCM also registered high ELISA readings (Figure S5C). This above threshold signal is likely caused by cross-reactivity of antibodies induced by exposure to the N protein of other coronaviruses with SARS-CoV-2 N and is consistent with high signal in negative control sera observed in other published results when testing for SARS-CoV-2 N antibodies.^4^^,^^28^

### Combined use of N and M antibody testing boosts the sensitivity of HCM and ELISA-based serological testing

Given the high prevalence of antibody responses to M observed by HCM, we examined whether the combined detection of antibodies to N and M could boost the sensitivity of SARS-CoV-2 serological testing in cases where S cannot be used to confirm prior SARS-CoV-2 infection, such as after vaccination. Five of 14 RT-PCR-confirmed SARS-CoV-2-positive samples that were N IgG negative by HCM had detectable M antibodies (Figure 2I). Three of these samples had sub-threshold N ELISA absorbances and were therefore also misclassified as SARS-CoV-2 negative by ELISA. Supplementing SARS-CoV-2 serological testing with screening for M antibodies by HCM therefore increased the number of detectable SARS-CoV-2 infections from 185/196 (94.4%) when using an N-based ELISA by itself, to 187/196 (95.4%) with combined testing for M IgG alongside N (Tables S3 and S4).

### SARS-CoV-2 M antibody kinetics contribute to enhanced serological sensitivity at later time points post infection

**Figure 3:**
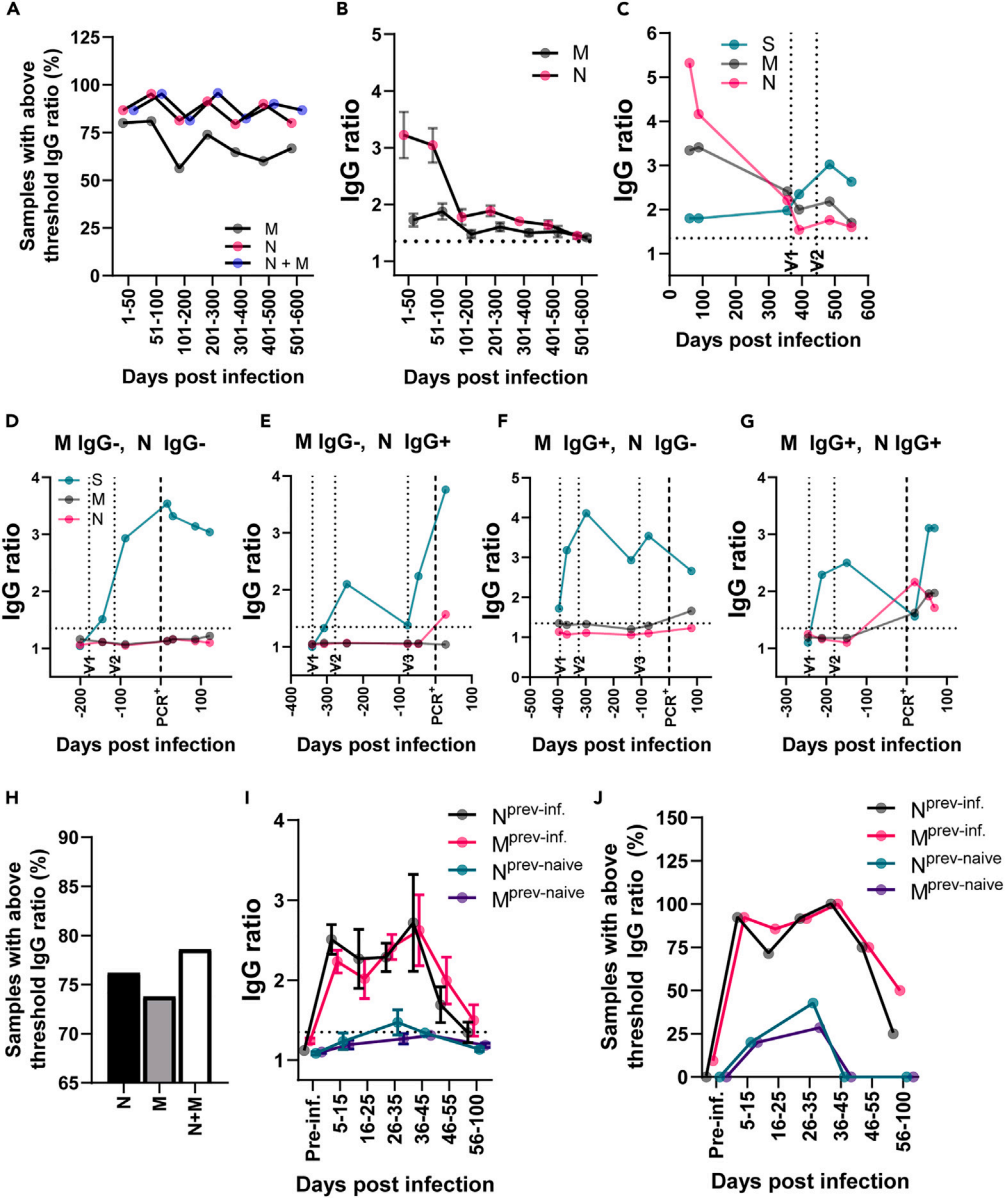
SARS-CoV-2 M antibodies induced by SARS-CoV-2 infection display characteristics that complement serological testing using N To analyze N and M antibody levels over time in SARS-CoV-2-infected samples from unvaccinated participants, SARS-CoV-2-positive samples collected from the same individuals at multiple time points post infection were assayed for antibodies against N or M by HCM. Ratios calculated for each sample were binned into groups spanning 100-day windows (except for samples obtained between days 1–50 and 51–100) depending on the time at which each serum sample was collected post infection. Number of samples analyzed per group: 1–50 (13), 51–100 (15), 101–200 (9), 201–300 (20), 301–400 (25), 401–500 (9), 501–600 (13). (A) Number of samples with above threshold antibody signal for N or M, or N and M combined at each time point. (B) Average IgG ratios in each group for N and M. Dashed line indicates the cutoff value for classification of samples as COVID-19 positive or negative. Error bars = S.E.M. (C) Representative plot of N, S, and M antibody levels over time from an unvaccinated individual infected with SARS-CoV-2, showing N antibody levels dropping below M by 356 days post infection. Dashed vertical lines labeled V1 and V2 indicate S-based vaccination dates. To assess M IgG responses relative to N in cases of breakthrough infection, N and M IgG levels were analyzed by HCM in 42 serum samples collected from individuals infected with SARS-CoV-2 after being vaccinated (Sample collection range: 10 and 80 days post infection, median collection time point: 29 days post infection). Representative plots of N, S, and M antibody levels over time from individuals infected with SARS-CoV-2 after being vaccinated showing examples of N and M antibody levels that were (D) undetectable after infection (E) positive for N IgG but negative for M IgG, (F) negative for N IgG but positive for M, or (G) positive for both N and M IgG. (H) Percentage of samples analyzed by HCM with above threshold N or M IgG ratios compared with the percentage of samples with above threshold IgG ratios when testing for N IgG is combined with M (N + M). (I) Longitudinal analysis of average N and M IgG levels over time in previously naive or previously infected individuals with SARS-CoV-2 infection following vaccination. Samples were binned into time points based on the time at which the serum was collected post infection. Number of samples analyzed per group (previously infected, previously naive): pre-infection (23, 9), 5–15 (13, 5), 16–25 (7, 0), 26–35 (12, 7), 36–45 (3, 1), 46–55 (4, 0), 56–100 (4, 4). Error bars = S.E.M. (J) Percentage of serum samples analyzed from previously naive and previously infected participants with above threshold N or M IgG responses at the indicated time points post infection. Pre-inf. = pre infection.

As N antibody levels have been reported to wane quickly after SARS-CoV-2 infection,^29^^,^^30^^,^^31^ we measured N and M IgG levels in plasma collected from individuals at multiple time points after SARS-CoV-2 infection, to establish whether M may be a better long-term marker of infection relative to N. While the sensitivity of serological testing using N antibodies as a marker remained higher than M at all time points we tested (Figure 3A), M antibody levels on average displayed a shallower time-dependent decline (Figures 3B and 3C). Screening for M IgG in samples collected between 500 and 600 days post infection increased the number of detectable SARS-CoV-2-specific IgG responses from 80% using N alone (12/15 positive) to 86.7% when testing for N and M together (13/15 positive). Further samples had detectable anti-M antibodies at 281 and 336 days after infection when no N IgG was detected (Figure 3A).

### SARS-CoV-2 M antibodies are a valuable serological marker of SARS-CoV-2 infection in vaccinated individuals

We investigated the utility of M relative to N as a marker of SARS-CoV-2 infection in 42 cases of infection in vaccinated individuals, collected between July 2021 and June 2022, therefore likely representing a mix of SARS-CoV-2 Delta and Omicron variant infections. The median time from positive RT-PCR to sampling was 29 days (interquartile range 21 days–33 days). Using HCM, antibodies to N could be detected in 76.2% (32/42) of all post-infection samples, with anti-M antibodies detected in 73.8% (31/42). The majority of samples analyzed were positive for both N and M IgG (71.5%, 30/42) (Figure S6A); however, as described for primary infections in unvaccinated individuals (Figure 2I), a number of samples were N IgG positive, M IgG negative (4.76%, 2/42) or N IgG negative, and M IgG positive (4.76%, 2/42) (Figures 3D–3G). As a result, the number of samples with detectable SARS-CoV-2-specific IgG increased from 76.2% or 73.8% using N or M alone, respectively, to 78.6% (33/42) by testing for both M and N IgG (Figure 3H). 71.4% (30/42) of participants also had evidence of SARS-CoV-2 infection prior to vaccination (classified as previously infected), whereas 28.6% (12/42) were infection naive prior to the post-vaccine infection. Average IgG ratios for both N and M were significantly lower in previously naive healthcare workers than those in previously infected individuals (Figures S6B and S6C). Antibodies against N or M were detectable in only 33.3% (4/12) and 25% (3/12) of previously naive individuals, respectively, compared with 87% (26/30) for N and 90% (27/30) for M in previously infected healthcare workers, demonstrating the boost to previously primed N and M antibody responses (Figure S6D). However, combined testing for M IgG alongside N in samples from previously naive individuals again increased the percentage of samples with detectable N- or M-specific antibodies to 41.7% (5/12) (Figure S6D).

Finally, we performed an analysis of antibody levels over time for N and M following infections in vaccinated individuals. Antibodies against N or M in previously naive individuals were close to or below threshold at all time points analyzed (Figures 3I, S7A, and S8). In contrast, average N and M antibody levels were above threshold at all time points in previously infected participants, peaking in samples collected between 36 and 45 days post infection, before generally undergoing a decline in samples collected after 45 days post infection (Figures 3I and S7B). The kinetics of anti-N and anti-M waning following infection in vaccinated individuals appeared to differ from the patterns observed following primary infections in unvaccinated individuals, with a more pronounced drop in levels after 50 days seen when infection occurred after vaccination (Figures 3B and S7C). This would suggest that following SARS-CoV-2 infection in vaccinated individuals, N and M IgG levels may wane quicker. However, for samples from previously infected individuals collected between 56 and 100 days post infection, a greater number could be classified as SARS-CoV-2 positive when using M IgG ratios instead of N (M: 50% (2/4), N: 25% (1/4)) (Figures 3J and S7B). Similar to the results described in Figures 3A–3C, M IgG after breakthrough infection may in some instances be sustained above the detectable threshold for longer than N IgG levels, thereby helping to detect additional SARS-CoV-2 infections. This sustained M response was evident in a number of previously infected patient IgG profiles where N IgG levels increased after infection before declining while M IgG levels at the same time points increased or remained constant (Figures 3G and S9 **- Profiles 1, 7, 12, and 16**).

## Discussion

The use of microscopy-based methods to detect serological responses to SARS-CoV-2 has to date remained relatively unexplored. A method to assay for SARS-CoV-2 antibodies by high-content microscopy has been described; however, the methodology used did not allow antibody responses against individual SARS-CoV-2 proteins to be assessed.^16^^,^^18^ In our study, we found the use of high-content microscopy to be an efficient method of screening patient sera for antibodies against individual SARS-CoV-2 proteins with sensitivity and specificity values close to an ELISA for N and S. Although the high-throughput, semi-quantitative method we present here is an upgrade on previous immunofluorescence-based serological screening methods, which have largely involved low-throughput qualitative assessment of antibody titers,^15^^,^^32^^,^^33^ manual inspection and scoring of captured images for detectable pathogen-specific antibodies remains a benefit of serological screening by HCM over other cell-based platforms such as flow cytometry. In addition, seeding of cell populations expressing viral proteins with distinct tags (i.e. M-FLAG expressing cells seeded with N-StrepTag expressing cells) in the same well can allow antibody levels against different proteins to be examined simultaneously in the same patient serum incubation, a benefit unique to cell-based screening platforms (Figure S10).

As demonstrated here, the deployment of an HCM-based platform to screen patient sera on a large scale is feasible; however, there are drawbacks to this system compared to ELISA-based approaches. HCM requires equipment to culture cells under sterile conditions and access to high-content microscopes which may not be available in low- to middle-income countries. We suggest that HCM-based serological screening would be of most value as a pandemic preparedness tool which would be used during the early stages of any future pandemic. The ability to rapidly identify the most antigenic components of a newly emergent pathogen would be extremely useful and could aid both in vaccine development and to help guide the selection of antigens for use in serological assays.

As part of this study, we initially took this approach and screened the whole proteome of SARS-CoV-2 looking for other serological targets in addition to N and S (data not shown). We only observed a strong serological response to the M protein which is consistent with other reports using cell-based systems.^21^ Interestingly, it has been reported that antibodies to multiple SARS-CoV-2 non-structural and accessory proteins can reproducibly be detected using other methods.^26^^,^^34^^,^^35^^,^^36^ At present, it is unclear why we fail to detect immunoreactivity to these proteins when they are expressed in cells. It is possible that immunofluorescence based detection of patient antibodies is not sensitive enough to detect the lower levels of antibodies produced against these proteins. Thus, we focused our attention on exploring the utility of the M protein for serological studies using HCM.

Recent studies investigating serum reactivity to the structural proteins of SARS-CoV-2 by flow cytometry have found that approximately 50%–60% of patients with COVID-19 contained antibodies against the SARS-CoV-2 M protein.^21^^,^^22^^,^^24^ In a further study using a biotinylated M peptide as antibody bait, SARS-CoV-2 M antibodies could be detected with 100% specificity in 62.3% (71/114) of patient serum samples at 3 months post infection.^23^ Our results indicate that SARS-CoV-2 M antibodies may be of even higher prevalence than previously appreciated as we could detect M antibody signal in close to 85% of PCR-validated SARS-CoV-2-positive samples. The higher seroprevalence observed in this study may be due to the fact that we are using the full-length M protein rather than just a short peptide (ELISA)^22^^,^^23^ or the surface-exposed portion of the protein (flow cytometry).^21^ These methods would only pick up antibodies targeting the extracellular region of M. In contrast, our assay will detect antibodies targeting both extracellular and intracellular regions of M. The higher seroprevalence of patients with M antibodies seen using HCM suggests that a significant portion of antibodies induced against M target intracellular portions of the M protein. Including these regions in antigens used in M immunoassays may therefore help to further enhance the sensitivity of serological testing against M.

The high seroprevalence of M antibodies in SARS-CoV-2-infected patients suggests that testing for M IgG may be of value to SARS-CoV-2 serology. The utility of M is underscored by complications associated with testing using N and S. The use of the S protein in vaccination programs means detection of S antibodies can no longer be used as an indicator of prior SARS-CoV-2 infection in vaccinated individuals who are subsequently infected with SARS-CoV-2. In addition, vaccinations combining N and S as immunogens to confer sterilizing immunity have also been proposed and tested^37^ which would preclude N- and S-based testing. A further complication relates to the specificity of IgG responses detected toward the SARS-CoV-2 N protein. Similar to other studies,^4^^,^^28^^,^^38^ we found here that 11% of pre-pandemic samples contained antibodies that cross-reacted with SARS-CoV-2 N but not S or M. Finally, rapid waning of N antibody levels may limit the use of N as a serological marker of SARS-CoV-2 infection at longer time points after infection.^29^^,^^30^^,^^31^

Our characterization of the SARS-CoV-2 M IgG response suggests that M possesses many characteristics that would complement serological testing using N. First, most N-positive samples were also positive for M and, in addition, a number of samples with low or undetectable N IgG could be unambiguously classified as SARS-CoV-2 positive when screening for infection using M. Second, despite the high degree of sequence similarity between M proteins of different coronaviruses, we observed no instances of serum cross-reactivity from our pre-pandemic (0/62) or seasonal coronavirus (0/6) sample sets with SARS-CoV-2 M, suggesting that serological testing using M may provide higher specificity than N. Third, M IgG often exhibited a shallower time-dependent decline than N. Longitudinal assessment of M antibody levels has indicated that M IgG remains stable and is detectable at 12 months post infection;^22^^,^^23^ however, a direct comparison with N antibody levels in the same samples over time has to our knowledge not been performed. Our results support the observations of previous studies,^22^^,^^23^ even showing that in many samples M IgG is detectable beyond 12 months post infection, and provide the first high resolution temporal profiles from individual patients of the SARS-CoV-2 M IgG response relative to N. These profiles highlighted instances where at later time points post infection M IgG levels remained stable and detectable while N IgG was undetectable or declining toward the detection threshold. Finally, all the above characteristics were largely mirrored in cases of infection in SARS-CoV-2-vaccinated individuals, particularly in participants with a prior history of SARS-CoV-2 infection, demonstrating the boosting of previously primed anti-N and M responses. We found vaccinated individuals with no prior history of SARS-CoV-2 infection rarely developed a detectable IgG response against N or M after a post vaccination SARS-CoV-2 infection, which is consistent with previous studies for N.^23^^,^^39^^,^^40^ Combined testing of patient sera against both antigens provided an increase in the number of samples with a detectable SARS-CoV-2-specific IgG response, however, again highlighting the advantages of dual N and M serological testing. For the above reasons, we propose that testing for M IgG by HCM or other means could in future be used to enhance the sensitivity and specificity of SARS-CoV-2 serological testing in both vaccinated and unvaccinated SARS-CoV-2-infected individuals. Recent structural studies of SARS-CoV-2 M have shown that it is feasible to make large quantities of a purified full-length M protein,^41^^,^^42^ suggesting it may be possible to develop an M ELISA which could be used for large-scale serological studies.

### Limitations of the study

Although this manuscript highlights the potential to use SARS-CoV-2 M IgG as a third high seroprevalence marker of infection, this is likely dependent on assaying against a full-length M protein as methods using extracellular regions of M to assay for M antibodies display lower sensitivity.^21^^,^^23^ As the use of a high-content microscope may not always be available, widespread use of M alongside N and S as part of ELISA-based sero-panels would be greatly facilitated by the ready availability of a purified full-length M protein. The production of a soluble full-length M protein for use in an ELISA was not addressed as part of this work but, as mentioned earlier, is likely to be possible given the success of other groups in producing a purified full-length M protein for use in structural studies.^41^^,^^42^

Additionally, while we speculate that the higher sensitivity of HCM-based anti-M testing is due to the ability of our HCM-based method to detect antibodies targeting the intracellular and extracellular regions of M, we did not test this hypothesis. It would therefore be of interest to examine whether the higher sensitivity of anti-M testing observed here is indeed due to a significant proportion of antibodies against M targeting intra-virion regions of the M protein. This would provide further evidence in favor of using a full-length M protein in serological studies.

The low numbers of samples used (42 serum samples) as part of this work examining the M antibody response following SARS-CoV-2 infection of vaccinated individuals is also a potential limitation. While this is to our knowledge the largest sample set from individuals with a breakthrough infection tested for an M antibody response, the conclusions drawn from this analysis would be boosted by testing an even larger number of serum samples obtained from individuals with a breakthrough infection. This may now be possible given a greater percentage of the population have now been vaccinated and likely subsequently infected with SARS-CoV-2.

## Consortia

PITCH consortium: Susanna Dunachie^7,8,9,10^, Paul Klenerman^7,8,11,12^, Eleanor Barnes^7,8,11,12^, Anthony Brown^7^, Sandra Adele^7,9^, Barbara Kronsteiner^7,9^, Sam M. Murray^7^, Priyanka Abraham^7^, Alexandra Deeks^7^, M. Azim Ansari^7^, Thushan de Silva^13,14^, Lance Turtle^15,16^, Shona Moore^18^, James Austin^18^, Alex Richter^17,18^, Christopher Duncan^19,20^, and Rebecca Payne^19^

## STAR★Methods

### Key resources table

**Table:** 

REAGENT or RESOURCE	SOURCE	IDENTIFIER
**Antibodies**		

StrepTactin-DY549	IBA Lifesciences	2-1565-050
Mouse anti-nucleocapsid	Genetex	Cat# GTX632269; RRID: AB_2888304
Mouse anti-strep-mAb	IBA lifesciences	Cat# 2-1507-001; RRID: AB_513133
rabbit anti-GAPDH	Proteintech	Cat# 60004-1-Ig; RRID: AB_2107436
rabbit anti-human-IgG AlexaFluor-488	Life technologies	A1101
HRP conjugated donkey anti-human IgG	Biolegend	410902
HRP conjugated anti-mouse	Jackson Immuno Research	115-035-008
HRP-conjugated goat anti-rabbit	Jackson Immuno Research	111-035-144
HRP conjugated goat anti-human IgG	Invitrogen	11594230

**Biological samples**		

Human serum	This study	N/A

**Chemicals, peptides, and recombinant proteins**		

SARS-CoV-2 Nucleocapsid	Dr Martin Nicklin, University of Sheffield	
SARS-CoV-2 Spike	Professor David James, University of Sheffield	

**Experimental models: Cell lines**		

HEK-293-T	ATCC	CRL-3216

**Recombinant DNA**		

pLVX-EF1alpha-IRES-puro SARS-CoV-2 N StrepTag	Professor Nevan Krogan, University of California San Francisco	Addgene ID: 141391
pLVX-EF1alpha-IRES-puro SARS-CoV-2 M StrepTag	Professor Nevan Krogan, University of California San Francisco	Addgene ID: 141386
pTwist-EF1alpha-IRES-puro S StrepTag	Professor Nevan Krogan, University of California San Francisco	NA
pcDNA6B-nCoV-M-FLAG	Dr James Edgar, University of Cambridge	NA

**Software and algorithms**		

Graphpad prism	GraphPad software	https://www.graphpad.com/ RRID:SCR_00279
Image Xpress Micro software package	Molecular Devices	https://www.moleculardevices.com/
Fiji	Schindelin et al.^43^	https://imagej.net/software/fiji/
Image Studio Lite	LICOR	https://www.licor.com/bio/image-studio/

**Other**		

DAPI	Merck	D9542
Saponin	Merck	84510-100G
Bovine serum albumin	Merck	BP1600-100
Prolong gold antifade	ThermoFisher scientific	P36930
PVDF membrane	Cytivia	10600023
4X Laemmli sample buffer	Biorad	1610747
DMEM	Merck	D6429-500ML
Fetal Bovine Serum (FBS)	Fisher Scientific	F4135-500ML
Penicillin-streptomycin	Merck	G1146-100ML
Nunc MaxiSorp	Thermo Scientific	442404
TMB substrate	KPL	5120-0074
HCl Stop solution	KPL	5150-0021
LICOR c-DiGiT imaging system	LICOR	https://www.licor.com/bio/cdigit/
Olympus BX61 motorised wide-field epifluorescence microscope	Olympus	NA
Molecular Devices Image Xpress Micro high content microscope	Molecular Devices	NA

### Resource availability

#### Lead contact

Further information and requests for resources and reagents should be directed to and will be fulfilled by the lead contact, Andrew Peden (a.peden@sheffield.ac.uk).

#### Materials availability

This study did not generate new unique reagents.

### Experimental models and study participant details

#### Recruitment and consent

Plasma samples used were from healthcare workers (HCWs) recruited at Sheffield Teaching Hospitals NHS Foundation Trust (STH) as part of the COVID-19 Humoral Immune Responses in front-line healthcare workers (HCWs) study (HERO), sampled in May and June 2020.^27^ Further longitudinal plasma samples from HCWs were used from a prospective, observational, cohort study (PITCH), where in Sheffield, participants were recruited under the Sheffield Teaching Hospitals (STH) Observational Study of Patients with Pulmonary Hypertension, Cardiovascular Disease and other Respiratory Disease (STH-Obs).^44^^,^^45^ Regulatory approval was provided by HRA and Health and Care Research Wales (HERO - 20/HRA/2180), and the Yorkshire and Humber – Sheffield Research Ethics Committee (STHObs - 18/YH/0441). Anonymised plasma samples from hospitalised COVID-19 patients (collected during February to May 2020) and plasma collected before 2017 during routine clinical care were used for assay validation purposes with approval from the STH R&D office as per standard practice. Plasma samples used for analysis of N and M antibody levels after infections in vaccinated individuals were obtained between July 2021 and June 2022.

#### Cell culture and transfections

HEK-293T cells (CRL-3216) originally obtained from ATCC, were grown in Dulbecco’s Modified Eagle Medium (Merck, D6429) supplemented with 10% fetal bovine serum (Gibco, 16140071), 100 IU/ml penicillin, 100 μg / ml streptomycin and 2mM glutamine (Merck, G1146-100ML). Cells were cultured at 37°C with 5% CO_2_ in a humidified incubator.

For pilot microscopy experiments, cells were seeded onto poly-l-lysine (PL) (Sigma, P8920) coated glass coverslips and left to adhere overnight at 37°C. The next day, plasmid DNA was mixed with transfection reagent (Fugene HD, E2311) at a ratio of 1 μg DNA : 3 μl Fugene and added to cells following a 10 minute incubation as per the manufacturer’s instructions. Cells were left at 37°C overnight before being processed for immunofluorescence microscopy.

### Method details

#### Antibodies and plasmids

StrepTagged proteins and human IgG were detected by microscopy using StrepTactin-DY549 (1:1000, IBA Lifesciences 2-1565-050) and rabbit anti-human-IgG AlexaFluor-488 (1:500, Life technologies A1101) respectively. Primary antibodies used for western blotting were mouse anti-SARS-CoV-2 nucleocapsid (Genetex, GTX632269), mouse anti-strep-mAb (1:1000, IBA lifesciences 2-1507-001) and rabbit anti-GAPDH (Proteintech, 60004-1-Ig). Secondary antibodies for western blotting were HRP conjugated donkey anti-human IgG (1:2000, Biolegend 410902), HRP conjugated anti-mouse (1:2000, Jackson Immuno Research 115-035-008) and HRP-conjugated goat anti-rabbit (1:2000, Jackson Immuno Research 111-035-144). StrepTagged SARS-CoV-2 N, S and M expression constructs were a kind gift from Professor Nevan Krogan, University of California. All SARS-CoV-2 proteins were human codon optimised and expressed from pLVX-EF1alpha-IRES-puro (N and M) or pTwist-EF1alpha-IRES-puro (S) plasmids as previously described.^8^

#### Immunofluorescence microscopy

Following transfection, coverslips were washed twice with phosphate buffered saline (PBS). Cells were then fixed for 15 minutes at room temperature with 4% paraformaldehyde (PFA; Park Scientific Limited, 04018-4) in PBS, PFA quenched with 100mM glycine (Fisher Scientific, 10070150) for 5 minutes and cells permeabilised and blocked with 0.1% Saponin (Sigma, S4521) and 1% bovine serum albumin (BSA) (Fisher Scientific, BP1600-100) diluted in PBS (IF buffer) for 10 minutes. For detection of intracellular proteins (N and M), coverslips were incubated with patient serum diluted at 1:125 in 50 μl of IF buffer for 1 hour at room temperature. The patient serum was then aspirated, and the coverslips were washed three times using IF buffer. For detection of cell surface S, coverslips were incubated with patient serum (1:125 in DMEM) for 1 hour at 37°C prior to fixation. Bound antibodies and StrepTagged viral proteins were detected by incubating the coverslips with an anti-human IgG secondary antibody conjugated to AlexaFluor-488 and StrepTactin-DY549 for 1 hour at room temperature. Cell nuclei were then counterstained with DAPI (Sigma, D9542), coverslips washed a further 3 times with IF buffer and mounted on microscope slides with Prolong Gold Antifade Mountant (ThermoFisher scientific, P36930). Patient samples were imaged using a 20x objective (Olympus BX61 motorised wide-field epifluorescence microscope) and images collected with a Hamamatsu Orca monochrome camera.

#### Manual quantification of immunofluorescence images

For manual quantification of bound antibodies recognising SARS-CoV-2 N and S proteins, average 488-fluorescence intensity was measured in FIJI^43^ from a minimum of six non-transfected and six transfected cells. Transfected cells were identified by the StrepTactin 549 staining. A ratio of cell associated fluorescence from non-transfected cells compared to transfected cells was obtained by dividing the 488-intensity measurements from transfected cells with the average 488-intensity from non-transfected cells. For manual quantification of anti-M antibody signal, 488-signal associated with Golgi localised M identified by the StrepTag staining was quantified from a minimum of six transfected cells. For non-transfected cells, 488-signal from the perinuclear region was measured and a ratio calculated as described above.

In our initial experiments assaying IgG responses against N, N antibody levels in patient sera were limiting as having too many transfected cells significantly reduced the anti-human IgG-1 Alexa-488 signal at higher transfected cell densities (Figure S1). To avoid using larger amounts of patient sera to increase the antibody specific Alexa-488 signal, we instead controlled the transfected to non-transfected cell ratio for N, S and M so that non-transfected cells were in excess (10:1 non transfected : transfected cells).

#### Automated high content microscopy and image analysis

HEK cells seeded in 6 well plates were transfected with SARS-CoV-2 plasmid DNA encoding either N, S or M proteins or mock transfected as described above. Cells were detached by rinsing with conditioned medium 24 hours after transfection. Mock transfected cells were mixed with transfected cells before being seeded into a poly-l-lysine coated 96 well plate (Greiner-bio-one, 655098) at a density of 30,000 – 40,000 cells per well and left to adhere overnight at 37°C.

The next day, cells were processed for immunofluorescence microscopy as described above. Images were acquired with a 20x objective on a Molecular Devices Image Xpress Micro high content microscope, collecting signal from DAPI, FITC and Texas red channels. A minimum of 4 non overlapping sites per well were imaged. Captured images were analysed using the Molecular Devices Image Xpress Micro software package. Transfected and non-transfected cells were identified as described in Figure 2. Total fluorescence signal for each cell identified by the software was divided by cell area and an average fluorescence intensity for transfected and non-transfected cells calculated from multiple cells per serum sample assayed.

#### Western blotting

HEK-293T cells expressing SARS-CoV-2 N grown in 6 well dishes were detached by rinsing with conditioned media and cell suspensions collected. Cells were then pelleted by centrifugation at 1000 *x g* for 10 minutes and the media then aspirated. Following one wash with PBS, the cell pellet was lysed in sample buffer containing 5% β-mercaptoethanol and boiled for 10 minutes at 95°C. Cell lysates were then loaded onto a Tris-Glycine polyacrylamide gel and proteins resolved by SDS-PAGE. Gels were transferred overnight via wet transfer in Tobin buffer (25 mM Tris, 192 mM glycine, 20% Methanol) onto PVDF membranes (Cytiva, 10600023) before blocking (PBS, 0.1% Tween-20 (Sigma, P7949) (PBST),5% skim powder milk (Sigma, 70166)) for 1 hour at room temperature. Membranes were then probed with patient sera diluted 1:1000 in blocking solution or an anti-StrepTag monoclonal antibody overnight. Membranes were then washed three times for 5 minutes each with PBST and incubated with donkey anti-human or anti-mouse HRP conjugated secondary antibodies for 1 hour. Following three 5-minute PBST washes, membranes were incubated with Clarity Western ECL substrate (Bio-rad, 170-5061) and signal detected using a LICOR c-DiGiT imaging system. Quantification of western blots was performed using the Image Studio Lite software package.

#### ELISA

ELISA for S and N proteins were performed as previously described.^27^ High binding microtiter plates (either Immulon 4HBX; Thermo Scientific, 6405, or Nunc MaxiSorp; Thermo Scientific, 442404) were coated overnight at 4°C with 50 μl/well SARS-CoV-2 protein diluted in PBS (pH 7.4). Either full-length spike produced in mammalian cells,^46^ or nucleocapsid produced in *E.coli* were used as antigens. Recombinant nucleocapsid protein was produced as previously described.^27^ Once coated, plates were washed 3x with 0.05% PBS-Tween, and blocked for 1 hour with 200 μl/well casein blocking buffer. Plates were emptied (no wash step) and loaded with 100 μl/well of samples and controls, diluted to 1:200 for a 2 hour incubation. Plates were washed 3x and loaded with 100 μl/well goat anti-human IgG-HRP conjugate (Invitrogen, 11594230) at 1:500 dilution for a 1 hour incubation. After a final 3x wash, plates were developed with 100 μl/well TMB substrate (KPL, 5120-0074) for 10 minutes in the dark. The reaction was stopped by addition of 100 μl/well 1% HCl stop solution (KPL, 5150-0021). Absorbances were read immediately at 450 nm.

### Quantification and statistical analysis

All graphs included in figures were made using the GraphPad Prism software package. Statistical comparisons between groups using an unpaired t-test and Pearson correlation coefficient analysis of N, S and M antibody relationships were all performed using GraphPad Prism. All experiments were performed a minimum of two times.

## Data Availability

• All data reported in this paper will be shared by the lead contact upon request. • This paper does not report original code. • Any additional information required to reanalyse the data reported in this paper is available from the lead contact upon request.

## Supplemental information

Document S1. Figures S1–S10 and Tables S1–S4

Document S2. PITCH consortium affiliations
