# Supplementary material for: Establishing SARS-CoV-2 membrane protein-specific antibodies as a valuable serological target via high-content microscopy
Source: iScience. 2023 Jun 7;26(7):107056. doi: 10.1016/j.isci.2023.107056 (PMC10246304; doi:10.1016/j.isci.2023.107056)
Supplement: Document S1. Figures S1–S10 and Tables S1–S4 [file mmc1.pdf]

iScience, Volume *26*

Supplemental information

Establishing SARS-CoV-2 membrane protein-specific antibodies as a valuable serological target via high-content microscopy

Daniel M. Williams, Hailey R. Hornsby, Ola M. Shehata, Rebecca Brown, Marta Gallis, Naomi Meardon, Thomas A.H. Newman, Megan Plowright, Domen Zafred, Amber S.M. Shun-Shion, Anthony J. Hodder, Deepa Bliss, Andrew Metcalfe, James R. Edgar, David E. Gordon, Jon R. Sayers, Martin J. Nicklin, Miles Carroll, PITCH Consortium, Paul J. Collini, Stephen Brown, Thushan I. de Silva, and Andrew A. Peden

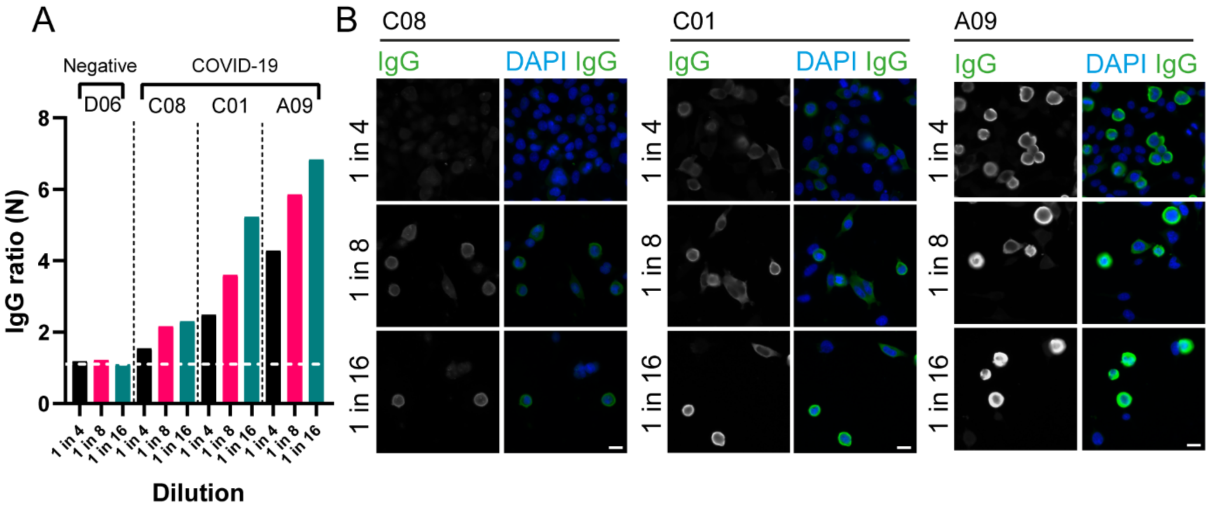

Supplementary Figure 1. Decreasing the number of transfected cells improves 488-intensity ratios. Related to Figure 1. **(A)** Automated quantification of 488 intensity ratios for COVID19 serum samples A09, C01 and C08 and the pre-pandemic negative serum sample D06. Decreasing cell density increases the IgG ratio for strong, intermediate and weak COVID-19 positive samples. **(B)** Representative images of StrepTagged SARS-CoV-2 N transfected cells mixed with non-transfected cells at a ratio of 1:10 and diluted as indicated. Serum samples with strong (A09), intermediate (C01) and weak (C08) SARS-CoV-2 N IgG responses were selected for imaging. Transfected and non-transfected cells were mixed together 24 hours after transfection and seeded into a 96 well plate at the indicated dilutions. The next day, cells were fixed, incubated with patient sera and processed for immunofluorescence. Scale bars = 20 µm.

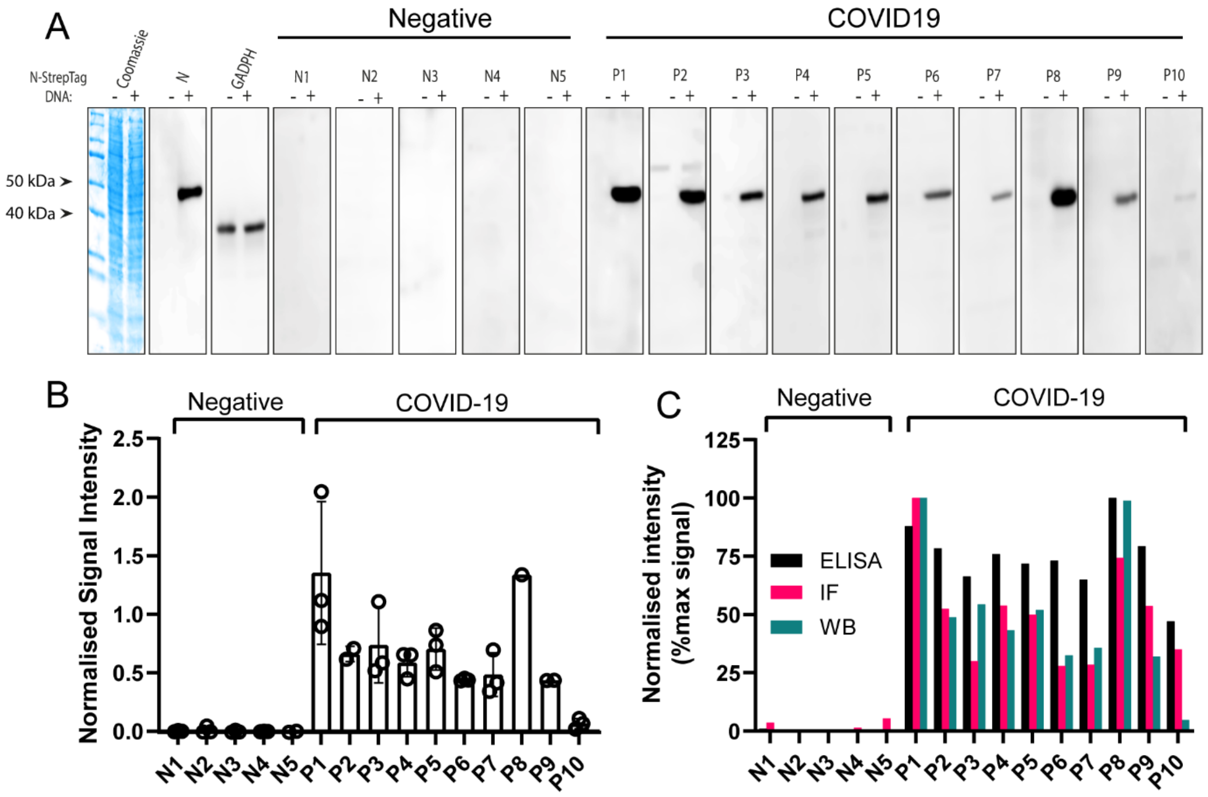

Supplementary Figure 2. Detection of SARS-CoV-2 N IgG by western blot. Related to Figure 1. **(A)** HEK-293T cell pellets transfected with either StrepTagged SARS-CoV-2 N (+) or empty vector (-) for 48 hours were lysed directly into SDS-PAGE sample buffer, lysates resolved by SDS-PAGE and transferred to PVDF. Individual strips of membrane were probed with sera from the sample set used in Figure 1 (N1-5, P1-10) and bound human IgG detected with HRP-labelled anti-human IgG secondary antibodies. **(B)** Quantification of signal detected by western blotting for each of the training set 15 serum samples. Minimum of N=2 for each sample. Not all serum samples were able to be analysed 3 times due to sample availability. **(C)** Comparison of normalised signal strength for each serum sample as measured by either western blotting, immunofluorescence or ELISA.

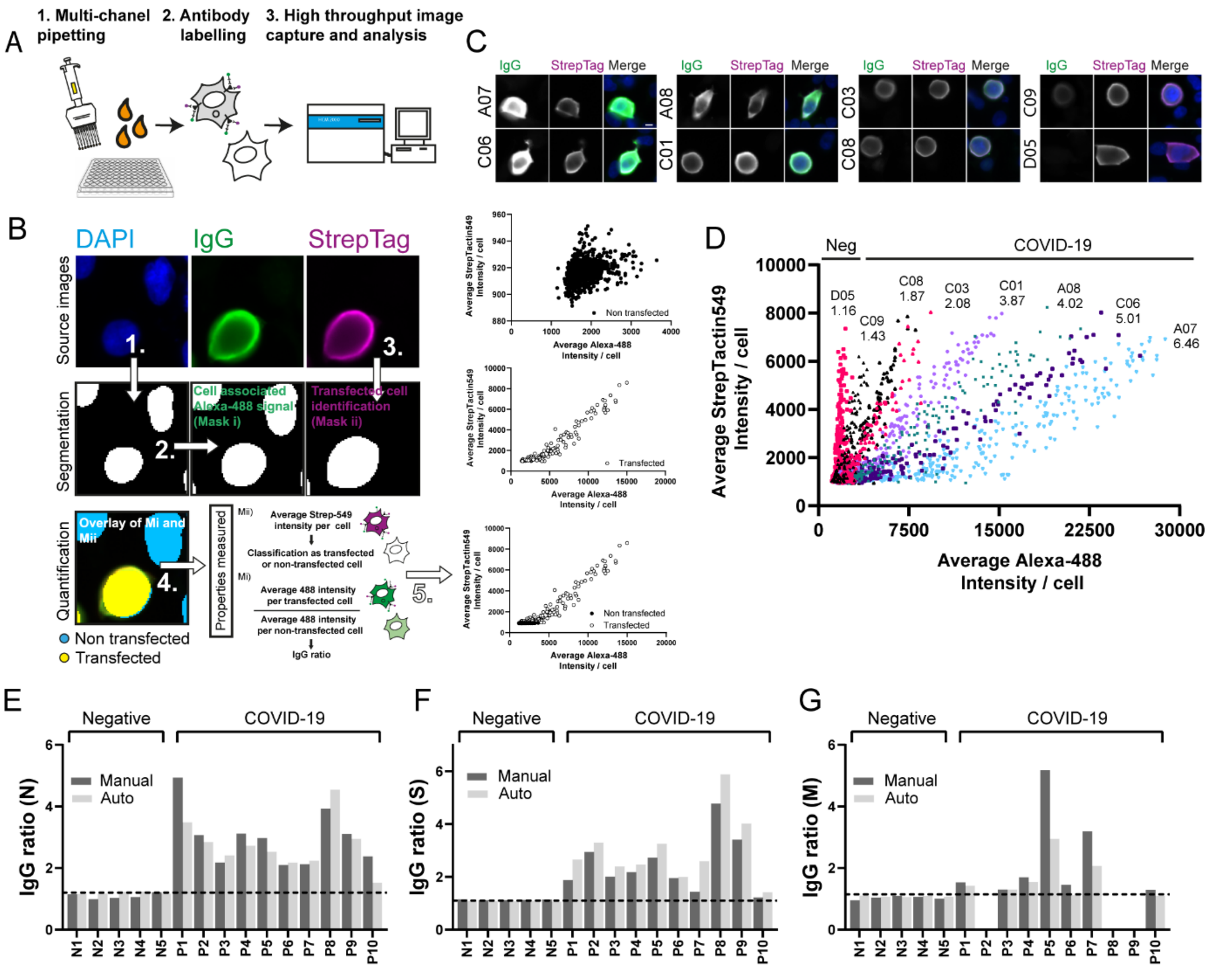

Supplementary Figure 3. Application of high content microscopy and automated image analysis to IF based SARS-CoV-2 antibody screening. Related to Figure 2. **(A)** Overview of workflow involved in processing samples for high content microscopy and automated image analysis and **(B)** summary of the method used by automated image analysis software to segment cells and classify as either transfected or non-transfected. Representative images are from cells transfected with StrepTagged-N and incubated with sera from a SARS-CoV-2 positive patient. Parameters measured by the software to calculate serum sample ratios are indicated after step 4. Representative scatter plots for transfected and non-transfected cells identified from a COVID-19 positive serum sample are shown after step 5. **(C)** Representative images of IgG signal associated with cells transfected with StrepTag-N following incubation with serum samples indicated in **(D).** Scale bar = 5 µm. **(D)** Scatter plots of Alexa-488 versus StrepTactin549 signal measured for StrepTag-N transfected cells identified by automated image analysis incubated with either pre-pandemic sera (D05) or SARS-CoV-2 IgG positive sera (C09, C08, C03, C01, A08, C06, A07). Plots are representative of the number of cells analysed from one field of view captured using a 20X objective with cells at approximately 60-80% confluency. SARS-CoV-2 N IgG positive serum samples range from weak positive to strong positive IgG responses. Comparison of 488 intensity ratios for **(E)** N, **(F)** S and **(G)** M IgG levels in pre-pandemic and COVID-19 serum samples generated by manual and automated image quantification for training set 15 samples previously described in Figure 1.

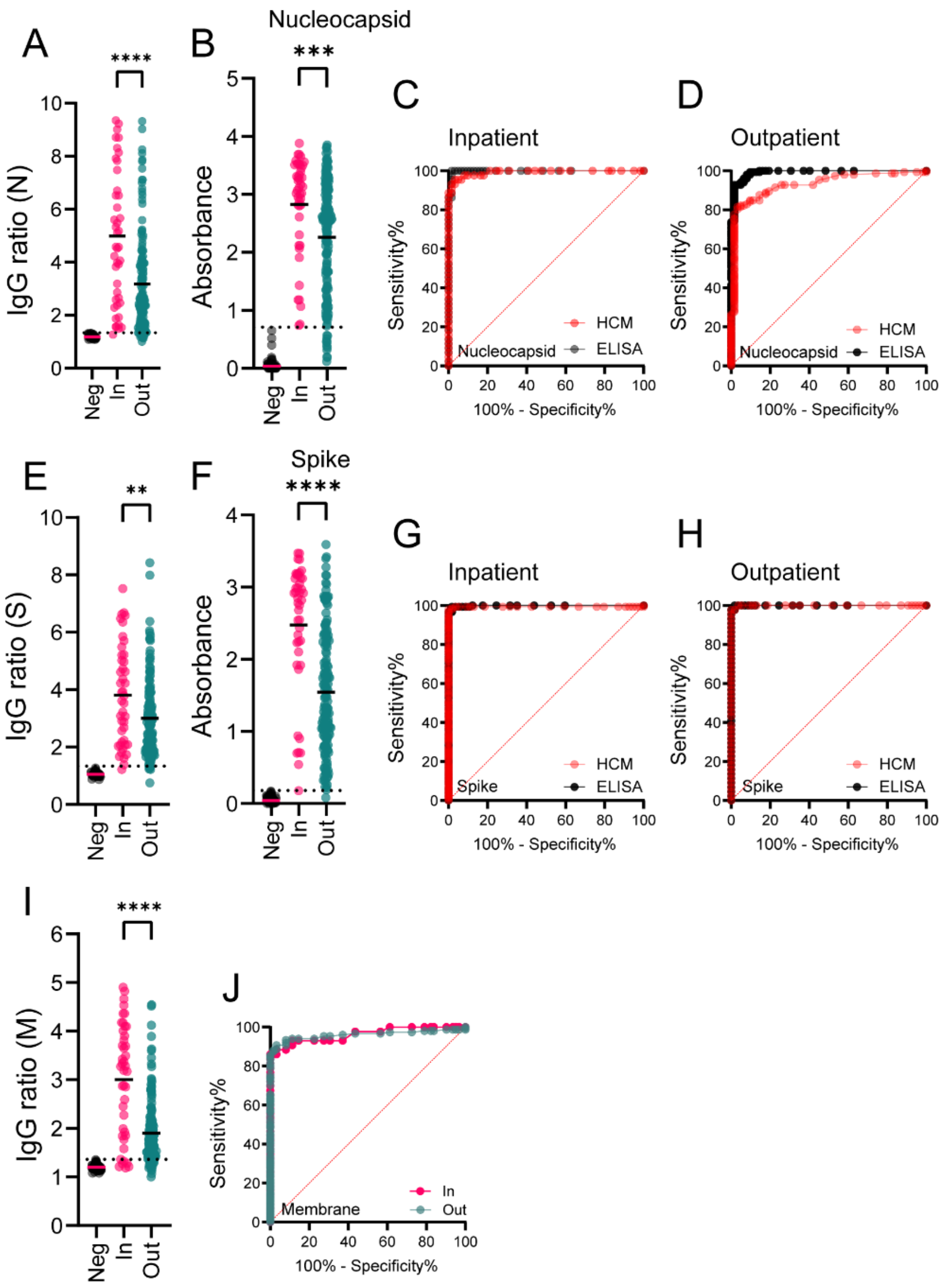

Supplementary Figure 4. Analysis of inpatient and outpatient IgG levels by HCM and ELISA. Related to Figure 2. Stratification of COVID-19 samples based on inpatient (n=44) and outpatient (n=152) status and comparison of HCM generated IgG ratios in inpatient and outpatient groups for **(A)** N **(E)** S and **(I)** M and ELISA absorbances in inpatient and outpatient groups for **(B)** N and **(D)** S. Dashed lines on the Y axis indicate the calculated HCM cut-off values for each antigen. ROC curves analysis of the performance of ELISA and HCM based detection of **(C-D)** N or **(G-H)** S IgG in SARS-CoV-2 infected inpatient or outpatient sample sets. **(J)** ROC curve analysis of M IgG ratios for inpatient and outpatient samples. ****P*<0.0001, ****P*<0.001 (unpaired t test).

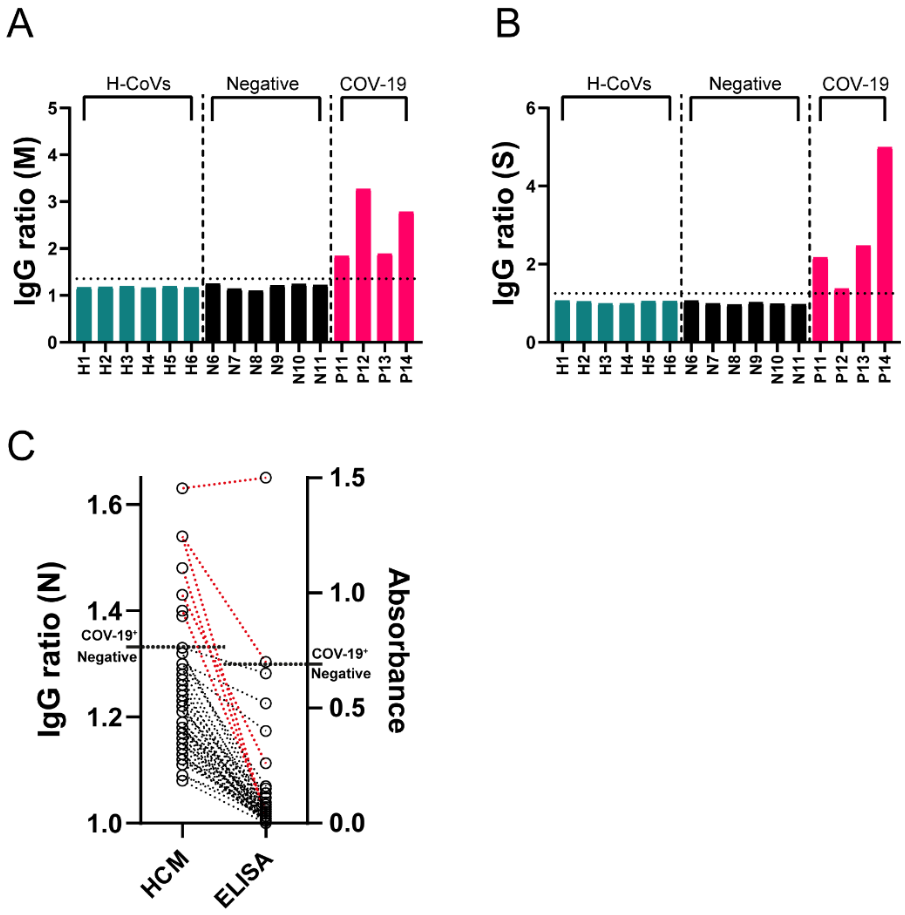

Supplementary Figure 5. SARS-CoV-2 S and M show no cross-reactivity with antibodies induced by seasonal H-CoVs. Related to Figure 2. Cross reactivity of sera taken from individuals infected with seasonal H-CoVs and **(A)** StrepTagged SARS-CoV-2 S and **(B)** StrepTagged SARS-CoV-2 M. No cross-reactivity of antibodies induced against seasonal H-CoVs can be seen with either S or M. **(C)** To examine whether high ELISA values were also found in the same pre-pandemic samples with seasonal coronavirus cross reactivity identified by HCM, IgG ratios and ELISA absorbances from these samples were plotted against one another. Dashed lines between data points link IgG ratios and ELISA absorbances from individual samples. Red dashed lines indicate samples with above threshold N signal identified by HCM. Half-length black dashed lines in the ELISA or HCM columns indicate the respective thresholds for classification of samples as COVID-19 positive or negative by ELISA or HCM.

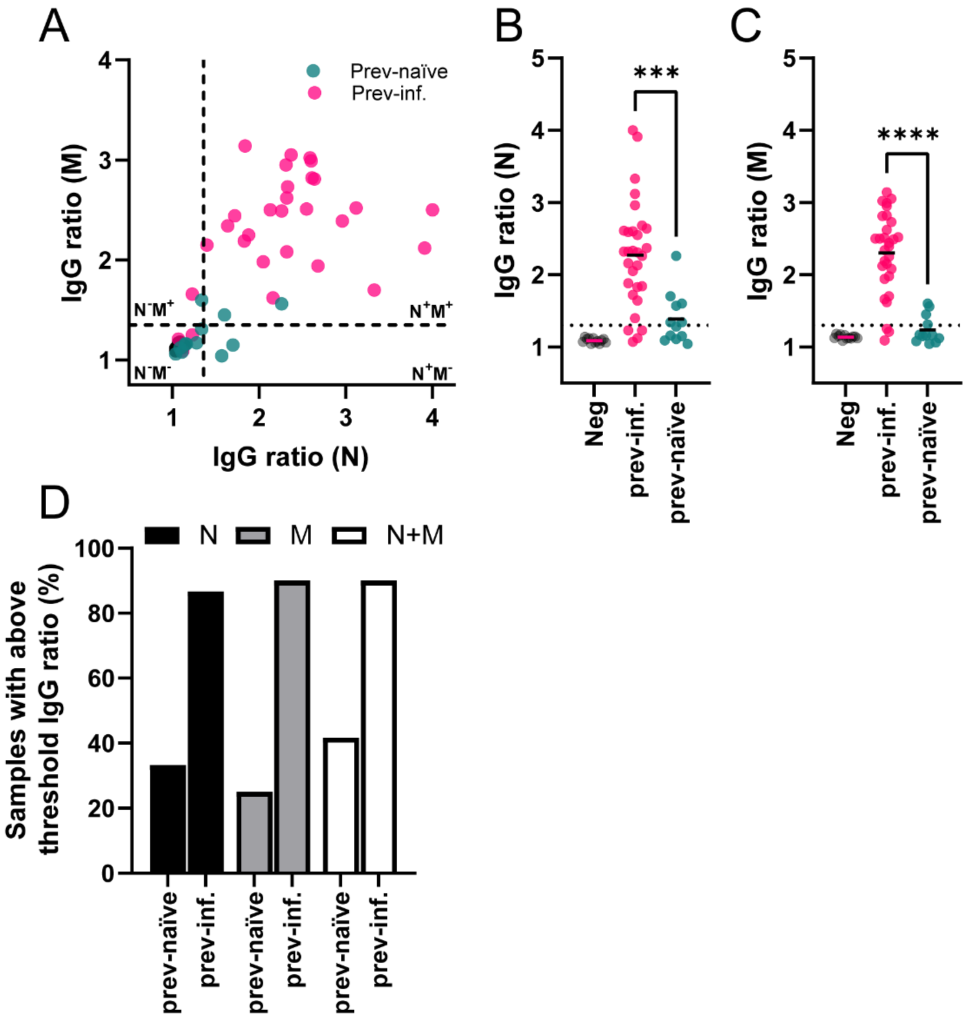

Supplementary Figure 6. HCM analysis of N and M antibody levels after infection in vaccinated previously-naive and previously-infected participants. Related to Figure 3. **(A)** Scatter plot of N and M IgG ratios from individual patient serum samples. Samples were stratified based on prior infection status with participants with no prior exposure to SARS-CoV-2 before being vaccinated classified as naïve, and those with an infection prior to vaccination classified as previously-infected (conv). Comparison of **(B)** N or **(C)** M IgG ratios analysed by HCM in pre-pandemic (Neg, n = 12) or breakthrough samples from previously-infected (n = 30) or previously-naïve (n = 12) participants. **(D)** Number of samples from previously-naïve or previously-infected individuals analysed with above threshold IgG responses for N or M alone, or N and M combined.

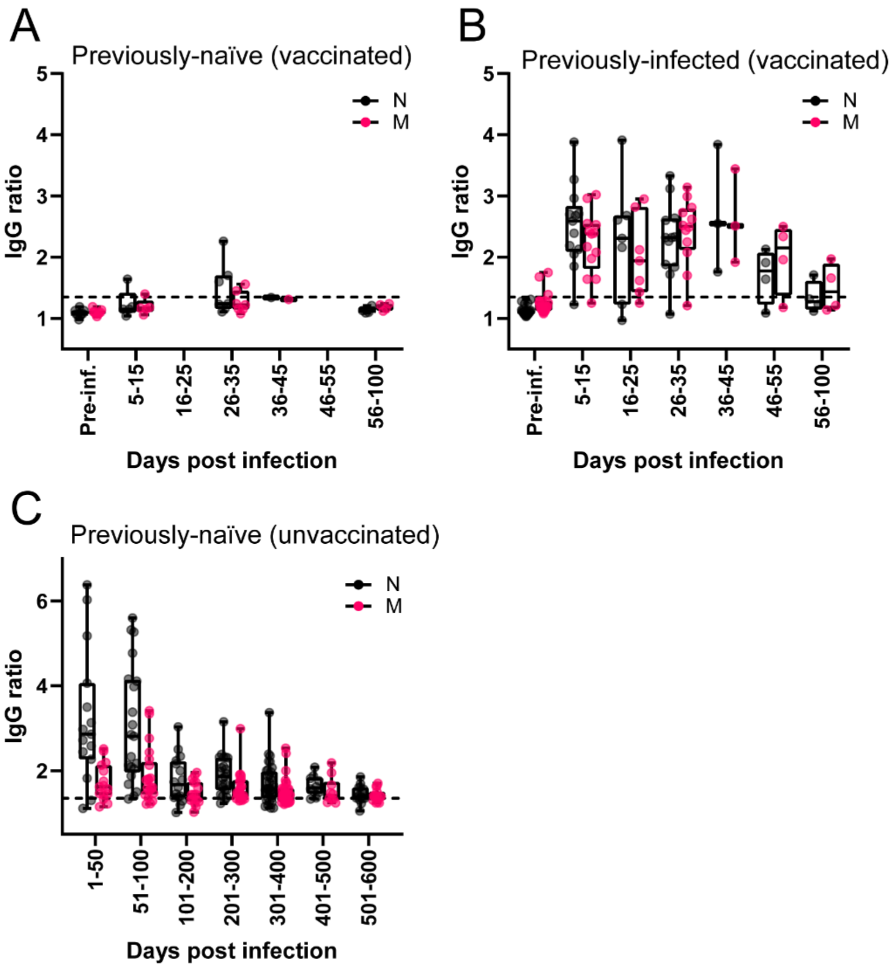

Supplementary Figure 7. Longitudinal analysis of N and M antibody levels in cases of breakthrough infection and cases of infection in unvaccinated participants. Related to Figure 3. IgG ratios for N and M were grouped into timepoints based on the time at which the serum was collected post infection and analysed by HCM. **(A)** Analysis of N and M antibody levels over time in samples obtained from participants infected after being vaccinated with no prior history of SARS-CoV-2 infection and **(B)** from participants with a prior SARS-CoV-2 infection (previously-infected). **(C)** Analysis of N and M antibody levels over time in samples obtained from unvaccinated participants infected with SARS-CoV-2. Boxes in box and whisker blots represent the upper and lower quartiles of values presented, with whiskers showing the highest and lowest values within each group.

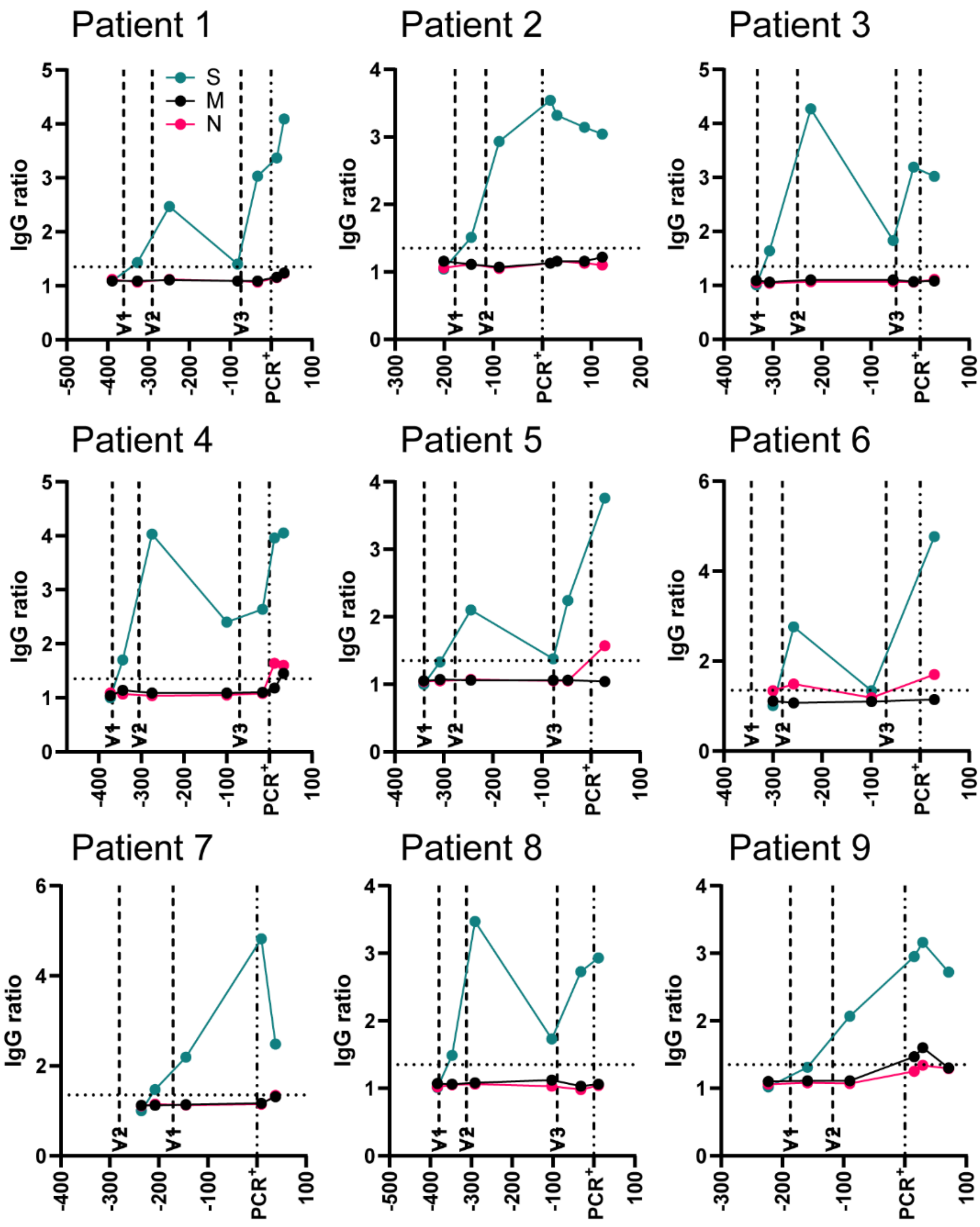

Supplementary Figure 8. Profiles from individual participants who were vaccinated and previously infection naïve showing N, S and M IgG levels over time after SARS-CoV-2 infection. Related to Figure 3. N, S and M IgG levels were measured overtime in naïve breakthrough samples by HCM and plotted against the timepoint at which they were collected relative to the date of breakthrough infection (indicated by PCR$^+$ on the x-axis). Vertical dashed lines over V1, V2 and V3 (marked on the x-axis) indicate the time of the first, second and third vaccination for each patient. Horizontal dashed line indicates the approximate cut-off value for classification of a sample as COVID-19 positive or negative.

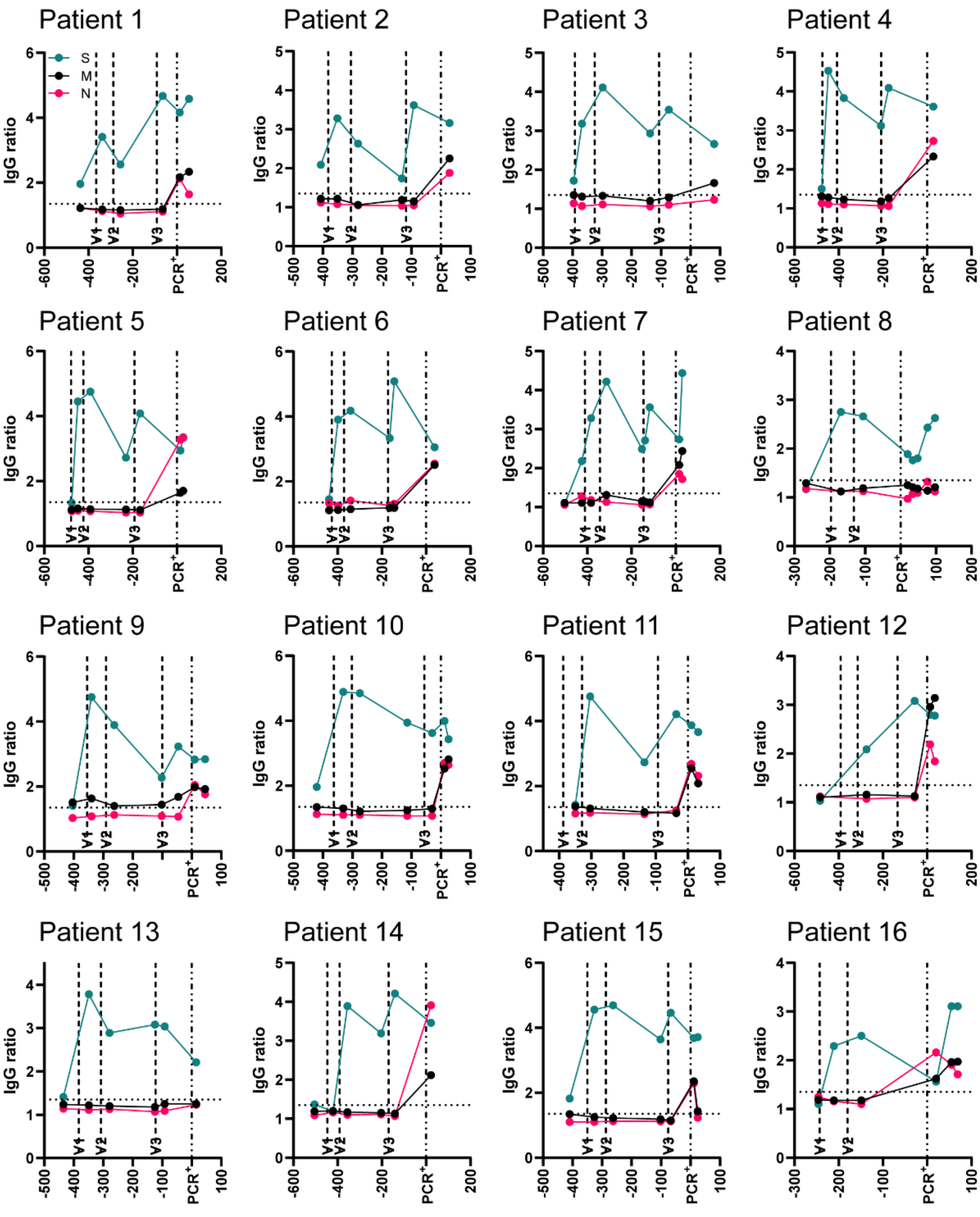

Supplementary Figure 9. Profiles from previously-infected individuals with re-infections following vaccination showing N, S and M IgG levels over time. Related to Figure 3. N, S and M IgG levels were measured over time by HCM and plotted against the timepoint at which they were collected relative to the date of breakthrough infection (indicated by PCR^{+} on the x-axis). Vertical dashed lines over V1, V2 and V3 (marked on the x-axis) indicate the time of the first, second and third vaccination for each patient. Horizontal dashed line indicates the approximate cut-off value for classification of a sample as COVID-19 positive or negative.

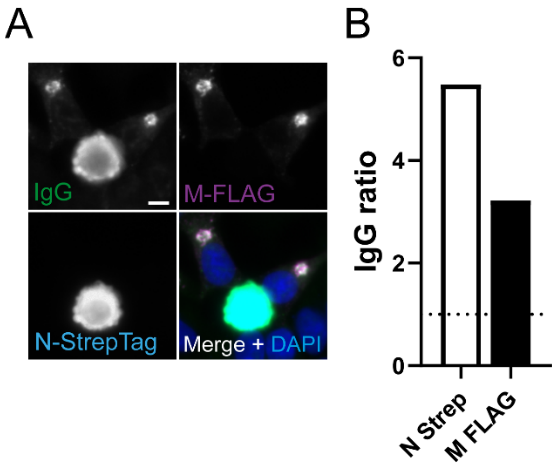

Supplementary Figure 10. Antibodies against N and M can be detected by HCM in the same serum staining using N and M constructs tagged with different epitopes. Related to Figure 2. **A)** Representative images of a mixed population of HEK-293T cells expressing either M-FLAG or N-StrepTag incubated with serum from a COVID-19 positive patient and imaged by HCM. Scale bar = 5 µm. **B)** Automated quantification of IgG signal associated with N StrepTag or M FLAG. Dashed line indicates cut off for classification of sample as COVID-19 negative or COVID-19 positive.

Supplementary Table 1 – Summary of ROC curve AUC values generated by ELISA or HCM for N and M. Related to Figure 2.

	HCM			ELISA		
	All patients	Inpatient	Outpatient	All patients	Inpatient	Outpatient
S	0.99	0.99	0.99	0.99	0.99	0.98
N	0.97	0.99	0.97	0.99	1.0	0.99
M	0.96	0.96	0.96	-	-	-

Supplementary Table 2 – Summary of sensitivity and specificity values for N, S, and M based cut-off values generated from HCM and ELISA ROC curves. Related to Figure 2.

		All patients		Inpatient		Outpatient	
		HCM	ELISA	HCM	ELISA	HCM	ELISA
S	Cut off value:	1.28	0.18	1.33	0.18	1.28	0.18
	Sensitivity:	97.9 (192/196)	99.5 (195/196)	97.9 (43/44)	100 (44/44)	98 (149/152)	99.3 (151/152)
	Specificity:	100	100	100	100	100	100
N	Cut off value:	1.34	0.71	1.41	0.72	1.34	0.71
	Sensitivity:	92.9 (182/196)	94.4 (185/196)	97.7 (43/44)	100 (44/44)	91.5 (139/152)	92.8 (141/152)
	Specificity:	100	100	100	100	100	100
M	Cut off value:	1.36	-	1.36	-	1.36	-
	Sensitivity:	84.7 (166/196)	-	86.1 (38/44)	-	84.9 (129/152)	-
	Specificity:	100	-	100	-	100	-

Supplementary Table 3 – Sensitivity and specificity values for HCM based detection of M and N individually, or M and N combined. Related to Figure 2.

		HCM Cut-off value	Sensitivity	Specificity	AUC
ALL	N	1.34	92.9 (182/196)	100	0.97
	M	1.35	84.7 (166/196)	100	0.96
	N + M	2.61	95.4 (187/196)	100	0.98
IN	N	1.41	97.7 (43/44)	100	0.99
	M	1.36	86.1 (38/44)	100	0.96
	N + M	2.73	97.4 (43/44)	100	0.99
OUT	N	1.34	91.5 (139/152)	100	0.97
	M	1.36	84.9 (129/152)	100	0.96
	N + M	2.61	94.7 (144/152)	100	0.98

Supplementary Table 4 – Sensitivity and specificity values based on combined detection of N antibodies by ELISA and detection of M antibodies by HCM. Related to Figure 2.

		SENSITIVITY	SPECIFICITY	CUT-OFF VALUES
ALL	N	94.4 (185/196)	100	0.71 ELISA
	N + M	95.4 (187/196)	100	0.71 ELISA, 1.36 HCM
IN	N	100 (44/44)	100	0.72 ELISA
	N + M	100 (44/44)	100	0.72 ELISA, 1.36 HCM
OUT	N	92.8 (141/152)	100	0.71 ELISA
	N + M	94.1 (143/152)	100	0.71 ELISA, 1.36 HCM
