# Supplementary material for: Establishing SARS-CoV-2 membrane protein-specific antibodies as a valuable serological target via high-content microscopy
Source: iScience. 2023 Jun 7;26(7):107056. doi: 10.1016/j.isci.2023.107056 (PMC10246304; doi:10.1016/j.isci.2023.107056)
Supplement: Document S2. PITCH consortium affiliations [file mmc2.pdf]

Susanna Dunachie[7,8,9,10], Paul Klenerman[7,8,11,12], Eleanor Barnes[7,8,11,12,] Anthony Brown[7], Sandra Adele[7,9], Barbara Kronsteiner[7,9], Sam M. Murray[7], Priyanka Abraham[7], Alexandra Deeks[7], M. Azim Ansari[7], Thushan de Silva[13,14], Lance Turtle[15,16], Shona Moore[18], James Austin[18], Alex Richter[17,18], Christopher Duncan[19,20] and Rebecca Payne[19]

[7]Peter Medawar Building for Pathogen Research, Nuffield Department of Clinical Medicine, University of Oxford, Oxford, UK. [8]Oxford University Hospitals NHS Foundation Trust, John Radcliffe Hospital, Oxford, UK. [9]Oxford Centre For Global Health Research, Nuffield Department of Clinical Medicine, University of Oxford, Oxford, UK. [10]Mahidol-Oxford Tropical Medicine Research Unit, Bangkok, Thailand. [11]Translational Gastroenterology Unit, University of Oxford, Oxford, UK. [12]NIHR Oxford Biomedical Research Centre, University of Oxford, Oxford, UK. [13]Department of Infection, Immunity and Cardiovascular Disease, University of Sheffield, Sheffield, UK. [14]Sheffield Teaching Hospitals NHS Foundation Trust, Sheffield, UK. [15]NIHR Health Protection Research Unit in Emerging and Zoonotic Infections, Institute of Infection, Veterinary and Ecological Sciences, University of Liverpool, Liverpool, UK. [16]Tropical and Infectious Disease Unit, Liverpool University Hospitals NHS Foundation Trust, Liverpool Health Partners, Liverpool, UK. [17]Institute of Cancer and Genomic Science, College of Medical and Dental Science, University of Birmingham, Birmingham, UK. [18]University Hospitals Birmingham NHS Foundation Trust, Birmingham, UK. [19]Translational and Clinical Research Institute Immunity and Inflammation Theme, Newcastle University, Newcastle, UK. [20]Department of Infection and Tropical Medicine, Newcastle upon Tyne Hospitals NHS Foundation Trust, Newcastle, UK.
